# Supplementary material for: SARS-CoV-2 spike protein predicted to form complexes with host receptor protein orthologues from a broad range of mammals
Source: Sci Rep. 2020 Oct 5;10:16471. doi: 10.1038/s41598-020-71936-5 (PMC7536205; doi:10.1038/s41598-020-71936-5)
Supplement: Supplementary file 7 — Supplementary Figure 7. [file 41598_2020_71936_MOESM7_ESM.pdf]

SARS-CoV-2 spike protein predicted to form complexes with host receptor protein orthologues from a broad range of mammals

Lam SD, Bordin N, Waman VP, Scholes HM, Ashford P, Sen N, van Dorp L, Rauer C, Dawson NL, Pang CSM, Abbasian M, Sillitoe I, Edwards SJL, Fraternali F, Lees JG, Santini JM, Orengo CA

Supplementary Figure S7

Animal photos courtesy of ENSEMBL and associated sources (https://www.ensembl.org/info/about/image_credits.html)

Primates

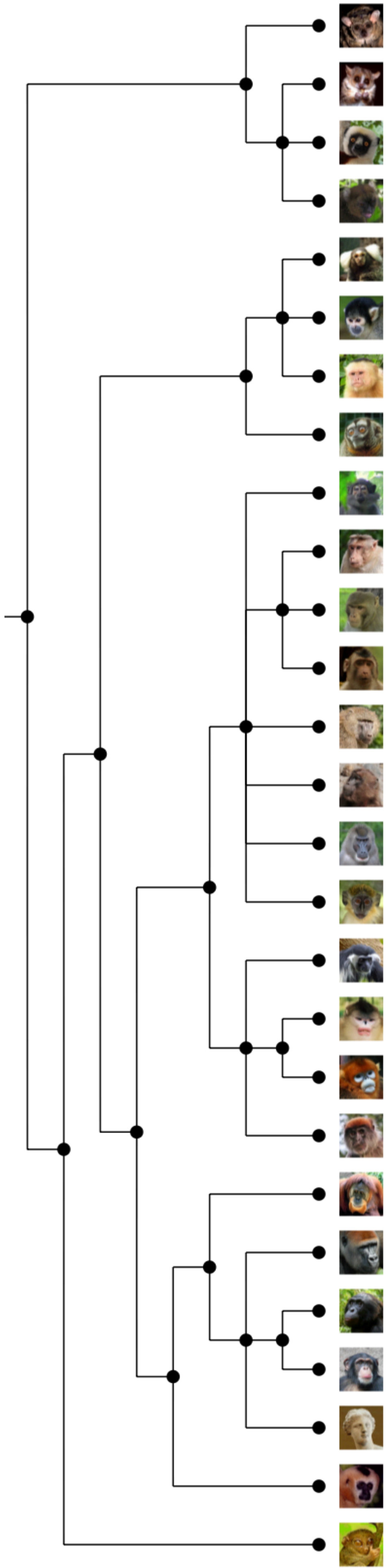

Animal Name	Protocol (2) - mCSM-PPI2 $\Delta\Delta G$	Sequence Identity	# Mutated Residue	Sum Grantham Score
Bushbaby	3.257	81.53	11	823
Mouse Lemur	2.821	82.22	8	727
Coquerel's sifaka	0.28	85.86	4	404
Greater bamboo lemur	1.127	84.85	4	239
**Marmoset**	3.438	87.44	4	280
Bolivian squirrel monkey	2.479	92.05	4	280
**Capuchin**	3.404	92.17	4	280
Ma's night monkey	2.932	92.17	4	280
Sooty mangabey	-0.243	95.21	1	5
**Crab-eating macaque**	0.166	95.21	1	5
**Macaque**	0.166	95.21	1	5
**Pig-tailed macaque**	-0.267	95.34	1	5
**Olive baboon**	-0.155	95.34	1	5
**Gelada**	-0.055	95.34	1	5
Drill	-0.297	94.96	1	5
Vervet-AGM	0.153	94.84	1	5
**Angola colobus**	0.013	95.88	2	107
Black snub-nosed monkey	NIL	NIL	NIL	NIL
**Golden snub-nosed monkey**	0.14	95.21	2	48
**Ugandan red Colobus**	0	95.03	0	0
**Orangutan**	0	97.39	0	0
**Gorilla**	0	99.01	0	0
Bonobo	0	99.01	0	0
**Chimpanzee**	0	99.01	0	0
**Human**	NA	NA	NA	NA
**Gibbon**	0.089	97.64	1	26
Tarsier	3.159	84.10	9	561

Rabbit and Rodents (1)

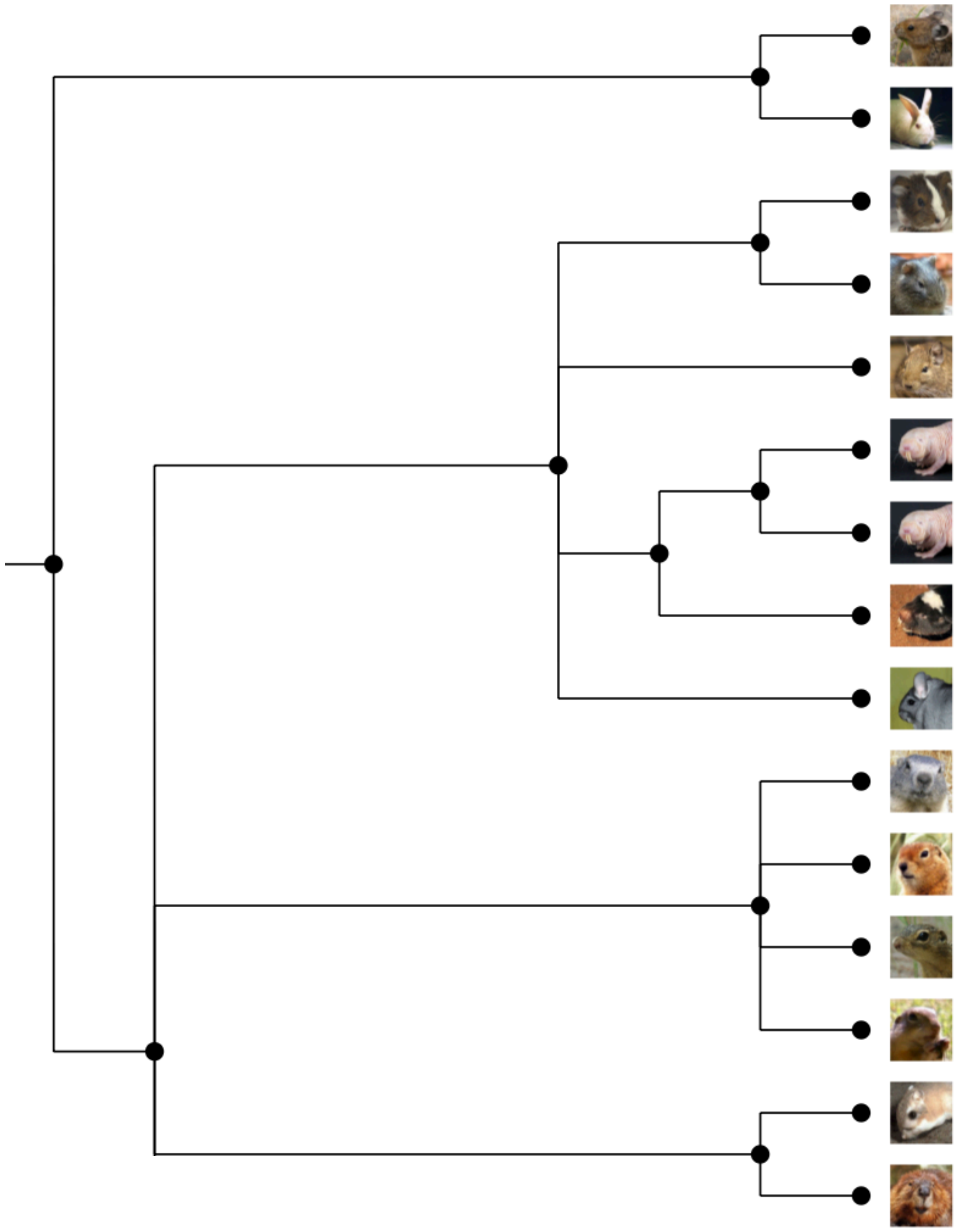

Animal Name	Protocol (2) - mCSM-PPI2 ΔΔG	Sequence Identity	# Mutated Residue	Sum Grantham Score
Pika	NIL	NIL	NIL	NIL
**Rabbit**	0.909	85.14	5	412
**Guinea Pig**	1.299	75.06	9	621
Brazilian Guinea Pig	NIL	NIL	NIL	NIL
Degu	1.953	84.47	8	629
Naked mole-rat male	1.065	84.47	6	493
Naked mole-rat female	1.065	84.60	6	493
Damara mole rat	1.84	84.72	7	578
Long-tailed chinchilla	3.339	84.72	9	688
Alpine marmot	1.833	84.88	7	557
Arctic ground squirrel	2.258	84.76	7	660
**Squirrel**	0.919	85.38	6	501
**Daurian ground squirrel**	0.876	84.88	6	501
Kangaroo rat	-0.391	82.12	5	390
American beaver	NIL	NIL	NIL	NIL

Rodents (2)

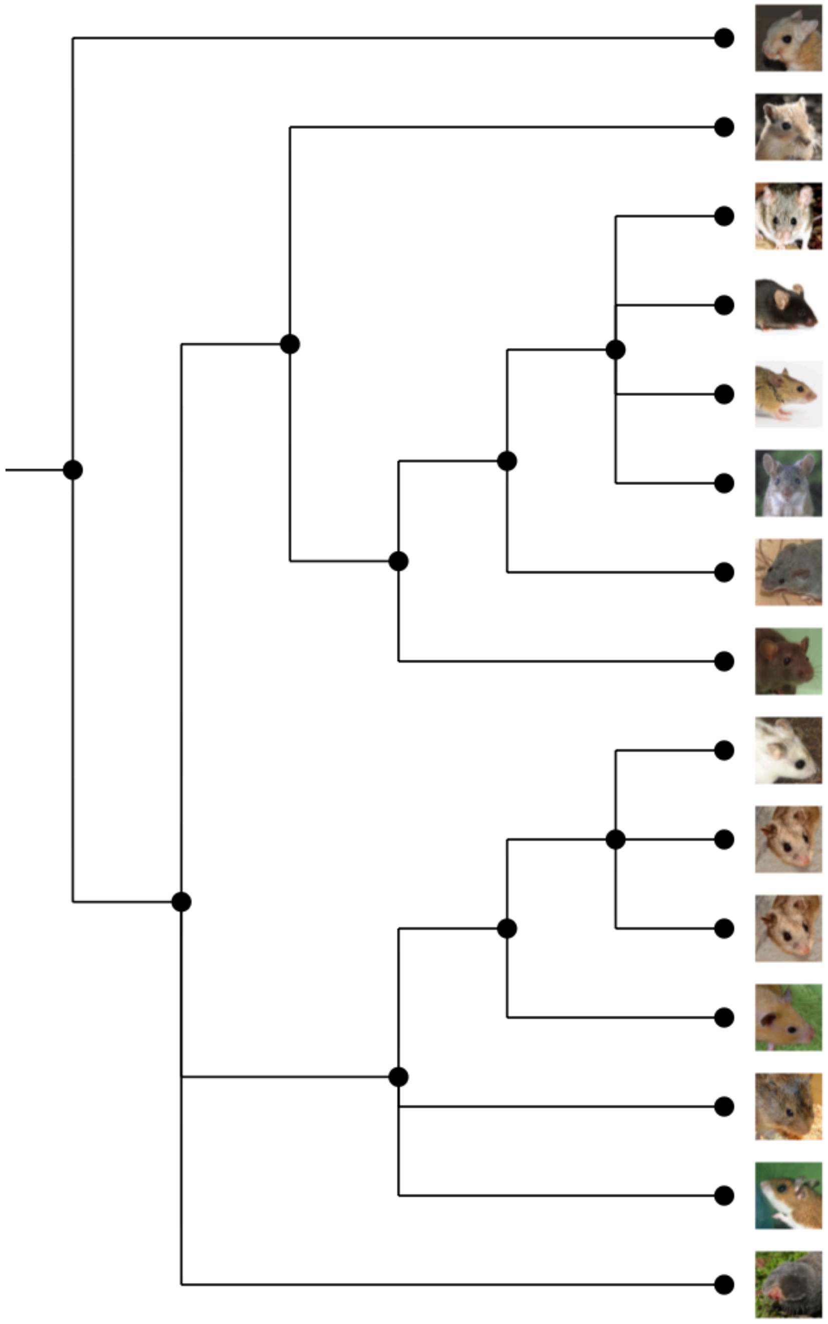

Animal Name	Protocol (2) - mCSM-PPI2 ΔΔG	Sequence Identity	# Mutated Residue	Sum Grantham Score
**Lesser Egyptian jerboa**	1.321	84.64	7	524
Mongolian gerbil	NIL	NIL	NIL	NIL
Ryukyu mouse	5.264	81.99	10	743
Mouse	5.552	81.86	11	837
Algerian mouse	3.653	82.37	10	785
Steppe mouse	4.556	78.34	11	841
Shrew mouse	4.611	82.87	10	750
Rat	5.947	82.37	13	818
**Chinese hamster CHOK1GS**	0.758	84.13	5	526
**Chinese hamster CRIGRI**	0.758	84.13	5	526
**Chinese hamster PICR**	0.758	84.01	5	526
**Golden Hamster**	0.42	84.26	4	428
Prairie vole	2.582	83.63	8	656
Northern American deer mouse	2.435	83.38	6	571
Upper Galilee mountains blind mole rat	0.281	82.37	4	381

Mammals (1)

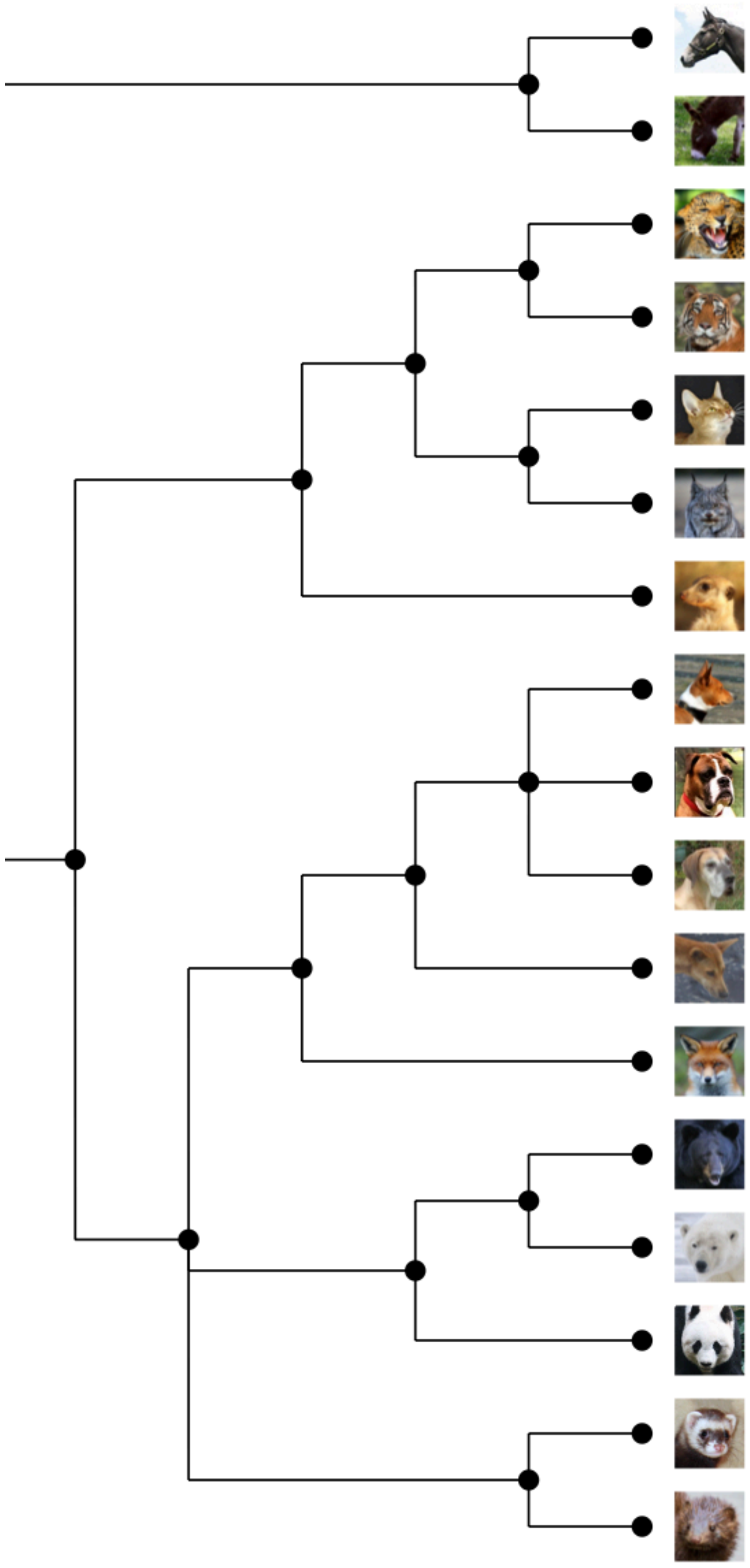

Animal Name	Protocol (2) - mCSM-PPI2 ΔΔG	Sequence Identity	# Mutated Residue	Sum Grantham Score
**Horse**	1.293	86.78	8	627
Donkey	1.293	86.90	8	627
**Leopard**	1.154	85.47	5	433
**Tiger**	NIL	NIL	NIL	NIL
**Cat**	1.472	84.85	5	433
**Canada lynx**	0.734	85.09	5	433
Meerkat	1.963	82.73	12	921
Dog - Basenji	NIL	NIL	NIL	NIL
**Dog**	0.446	84.14	6	516
Dog - Great Dane	0.446	84.14	6	516
Dingo	-0.136	84.01	6	516
**Red fox**	1.77	83.63	7	610
**American black bear**	0.044	84.01	7	493
**Polar bear**	0.836	82.89	7	493
**Panda**	0.882	83.19	7	493
**Ferret**	1.049	82.74	11	827
**American mink**	0.632	83.00	11	800

Mammals (2)

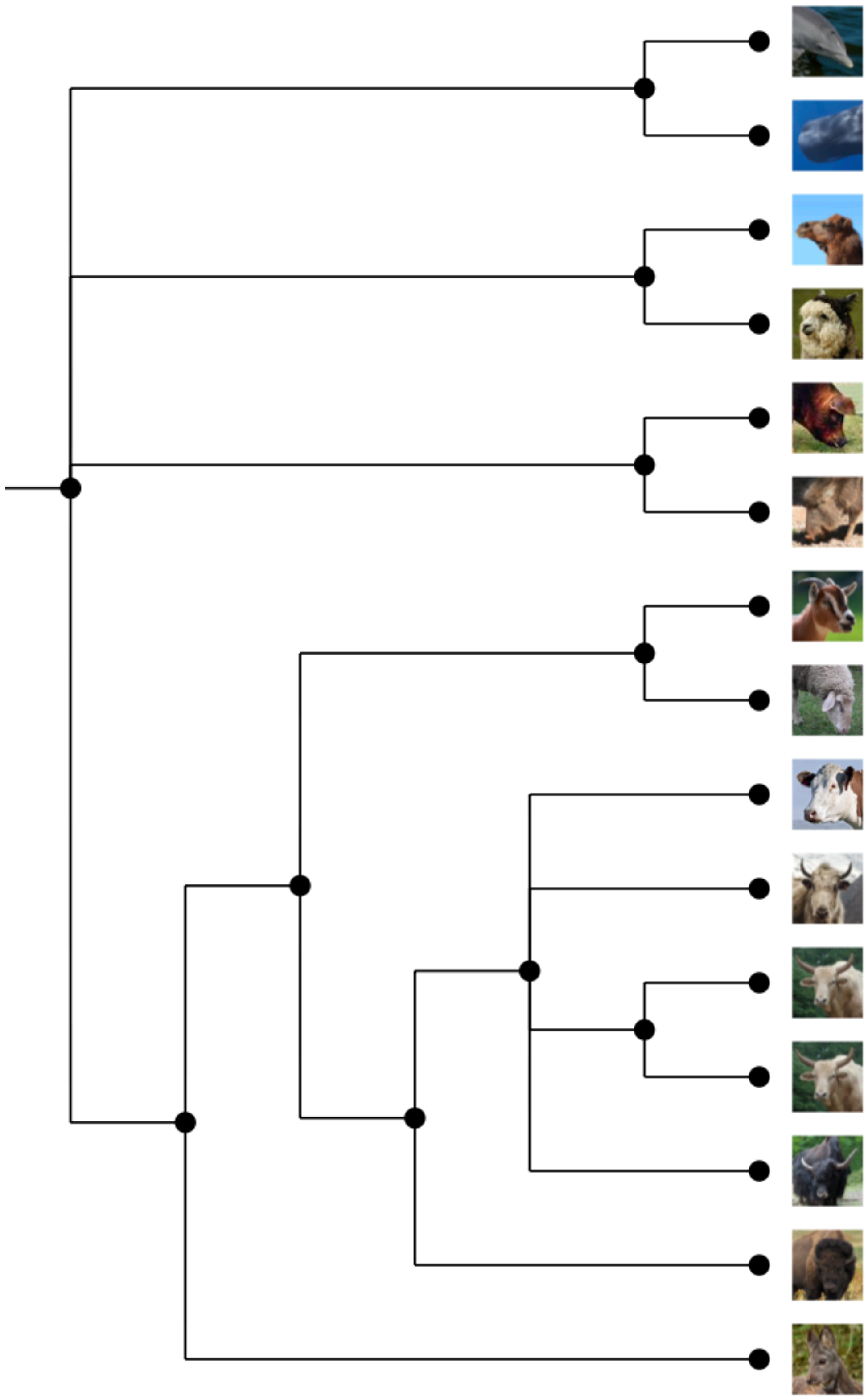

Animal Name	Protocol (2) - mCSM-PPI2 ΔΔG	Sequence Identity	# Mutated Residue	Sum Grantham Score
**Dolphin**	1.399	75.65	9	548
**Sperm whale**	0.784	82.73	7	563
**Arabian camel**	0.94	83.23	8	634
Alpaca	NIL	NIL	NIL	NIL
**Pig**	1.77	81.74	8	514
Chacoan peccary	1.416	81.99	10	720
**Goat**	1.165	81.62	7	467
**Sheep**	-0.005	81.64	7	470
**Cow**	0.56	81.12	7	470
**Domestic yak**	0.56	81.48	7	470
Hybrid - Bos Taurus	NIL	NIL	NIL	NIL
**Hybrid - Bos Indicus**	0.56	81.48	7	470
**Wild yak**	0.56	81.37	7	470
American bison	NIL	NIL	NIL	NIL
Siberian musk deer	-0.349	81.24	7	470

Mammals (3)

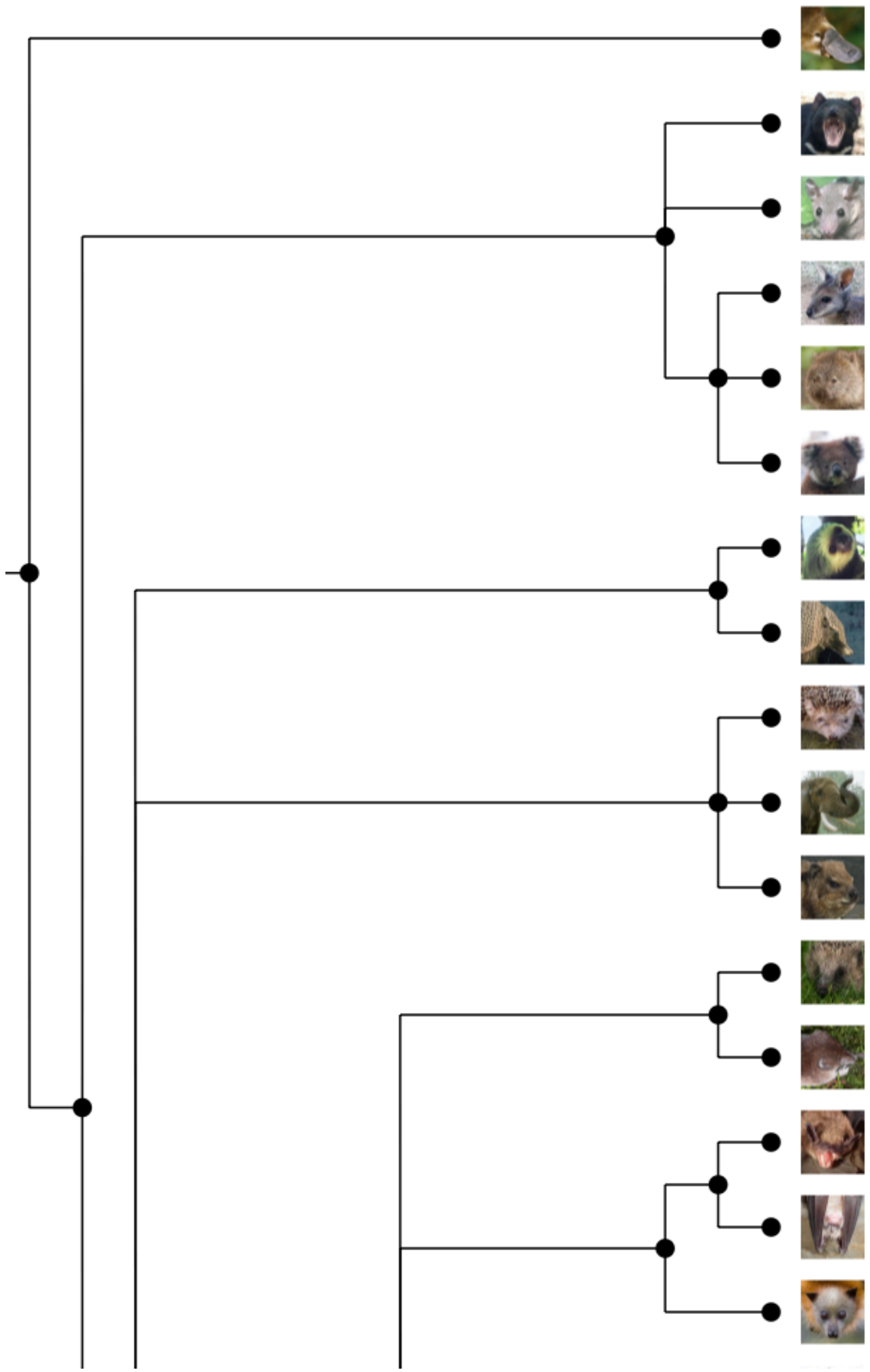

Animal Name	Protocol (2) - mCSM-PPI2 ΔΔG	Sequence Identity	# Mutated Residue	Sum Grantham Score
Platypus	4.083	68.14	17	1092
Tasmanian devil	NIL	NIL	NIL	NIL
Opossum	5.332	71.20	16	1094
Wallaby	NIL	NIL	NIL	NIL
Common wombat	2.751	71.68	16	870
Koala	2.503	70.65	15	848
Sloth	NIL	NIL	NIL	NIL
Armadillo	5.364	78.71	16	981
Lesser hedhehog tenrec	NIL	NIL	NIL	NIL
Elephant	3.059	80.50	9	648
Hyrax	NIL	NIL	NIL	NIL
Hedgehog	3.858	81.98	16	1033
Eurasian common shrew	6.465	69.14	19	1213
Microbat	4.429	80.45	15	1050
**Greater horseshoe bat**	3.723	81.36	14	981
Megabat	1.962	79.75	9	374

Birds (1)

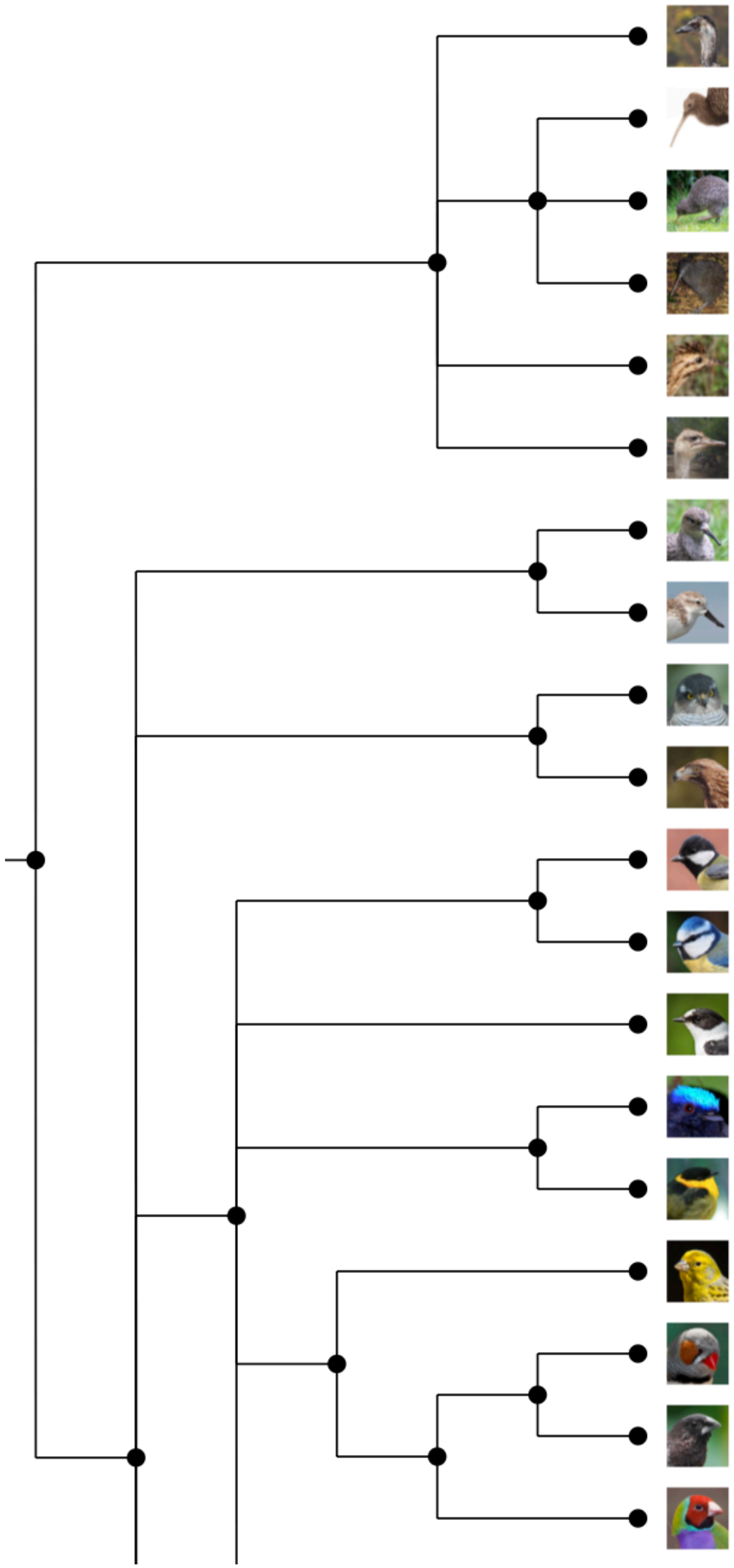

Animal Name	Protocol (2) - mCSM-PPI2 ΔΔG	Sequence Identity	# Mutated Residue	Sum Grantham Score
Emu	6.607	65.83	20	1449
Great spotted kiwi	4.371	66.17	21	1613
Little spotted kiwi	4.521	66.29	21	1613
Okarito brown kiwi	5.151	66.42	21	1613
Chilean tinamou	2.884	63.81	19	1256
African ostrich	4.439	62.27	20	1484
Ruff	5.231	63.67	22	1573
Spoon-billed sandpiper	5.232	66.00	21	1431
Eurasian sparrowhawk	4.157	66.42	20	1299
Golden eagle	3.902	66.00	21	1240
Great Tit	2.688	67.37	22	1330
Blue tit	1.267	67.37	20	1193
Flycatcher	6.546	66.58	20	1186
Blue-crowned manakin	4.996	66.25	23	1507
Golden-collared manakin	5.898	65.84	23	1507
Common canary	3.786	64.89	19	1127
Zebra finch	7.275	65.34	20	1182
Bengalese finch	3.109	64.32	20	1174
Gouldian finch	2.603	66.54	21	1304

Birds (2)

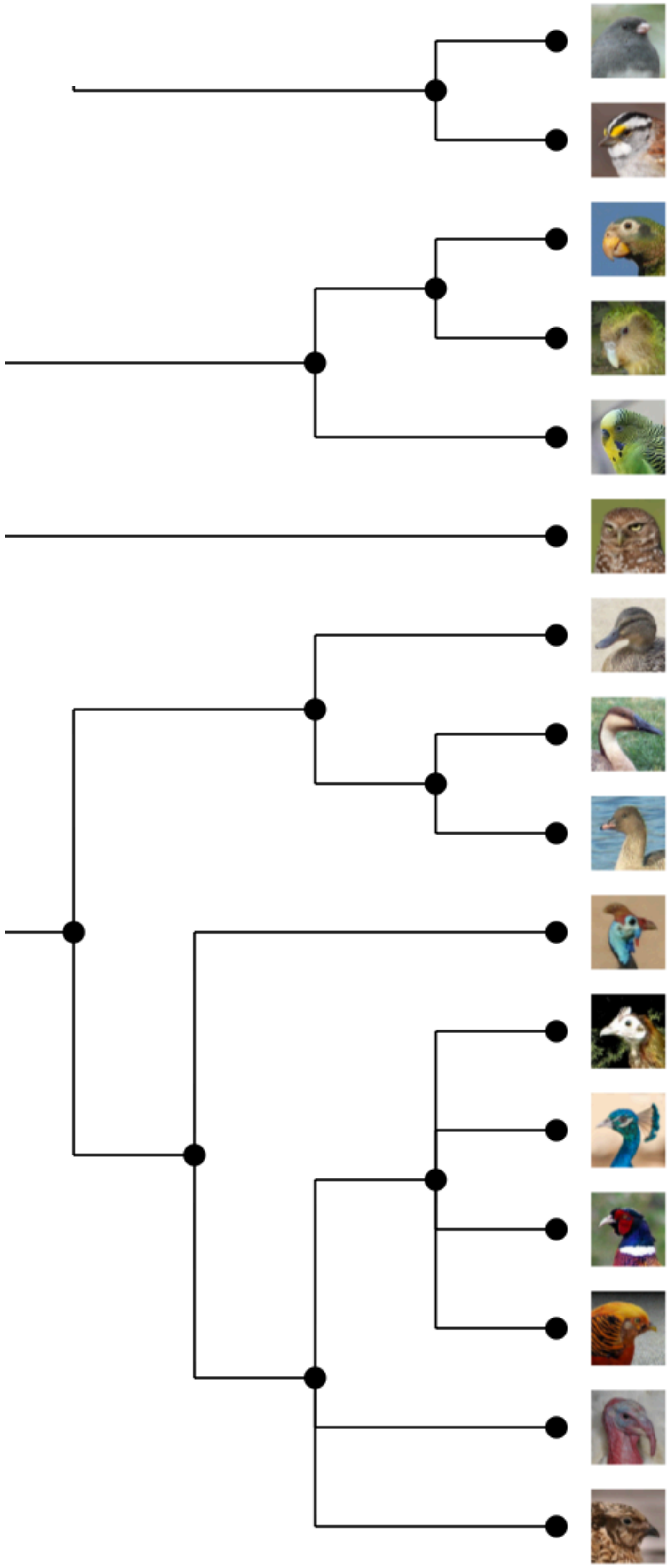

Animal Name	Protocol (2) - mCSM-PPI2 ΔΔG	Sequence Identity	# Mutated Residue	Sum Grantham Score
Dark-eyed junco	5.861	66.46	20	1338
White-throated sparrow	4.551	65.88	21	1381
Yellow-billed parrot	4.227	66.50	20	1376
Kakapo	6.433	61.78	21	1278
Budgerigar	3.885	62.67	20	1343
Burrowing owl	5.064	65.79	19	1287
Duck	5.889	69.29	18	1394
Swan goose	3.604	65.00	17	1173
Pink-footed goose	5.428	65.06	19	1437
Helmeted guineafowl	5.791	65.35	20	1439
Chicken	5.001	65.97	19	1350
Indian peafowl	NIL	55.84	NIL	NIL
Ring-necked pheasant	5.694	65.26	19	1414
Golden pheasant	NIL	62.41	NIL	NIL
Turkey	5.731	64.20	19	1412
Japanese quail	4.377	66.79	20	1490

Reptiles / Amphibian

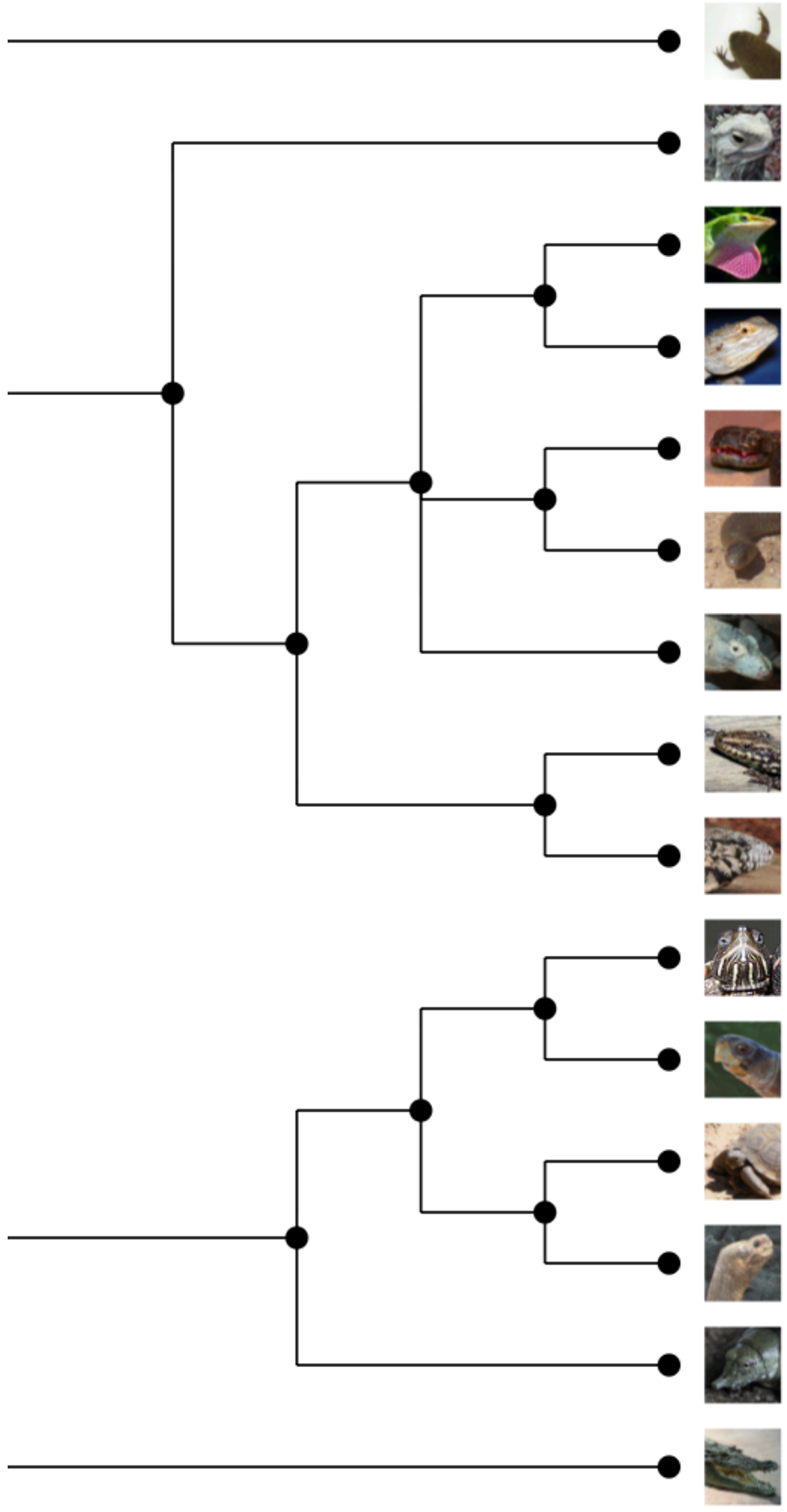

Animal Name	Protocol (2) - mCSM-PPI2 ΔΔG	Sequence Identity	# Mutated Residue	Sum Grantham Score
Tropical clawed frog	3.883	60.15	22	1458
Tuatara	2.656	64.04	16	1232
Anole lizard	5.411	63.66	22	1567
Central bearded dragon	4.993	64.87	21	1525
Mainland tiger snake	8.032	61.61	20	1338
Eastern brown snake	5.643	59.50	20	1280
Komodo dragon	5.32	61.31	22	1731
Common wall lizard	NIL	59.09	NIL	NIL
Argentine black and white tegu	6.117	65.05	23	1501
Painted turtle	3.616	66.46	21	1583
Three-toed box turtle	4.223	66.96	21	1583
Agassiz's desert tortoise	NIL	62.27	NIL	NIL
Abingdon island giant tortoise	4.756	66.75	22	1605
Chinese softshell turtle	4.48	67.12	21	1556
Australian saltwater crocodile	2.533	65.63	21	1463

Fishes (1) and Shark

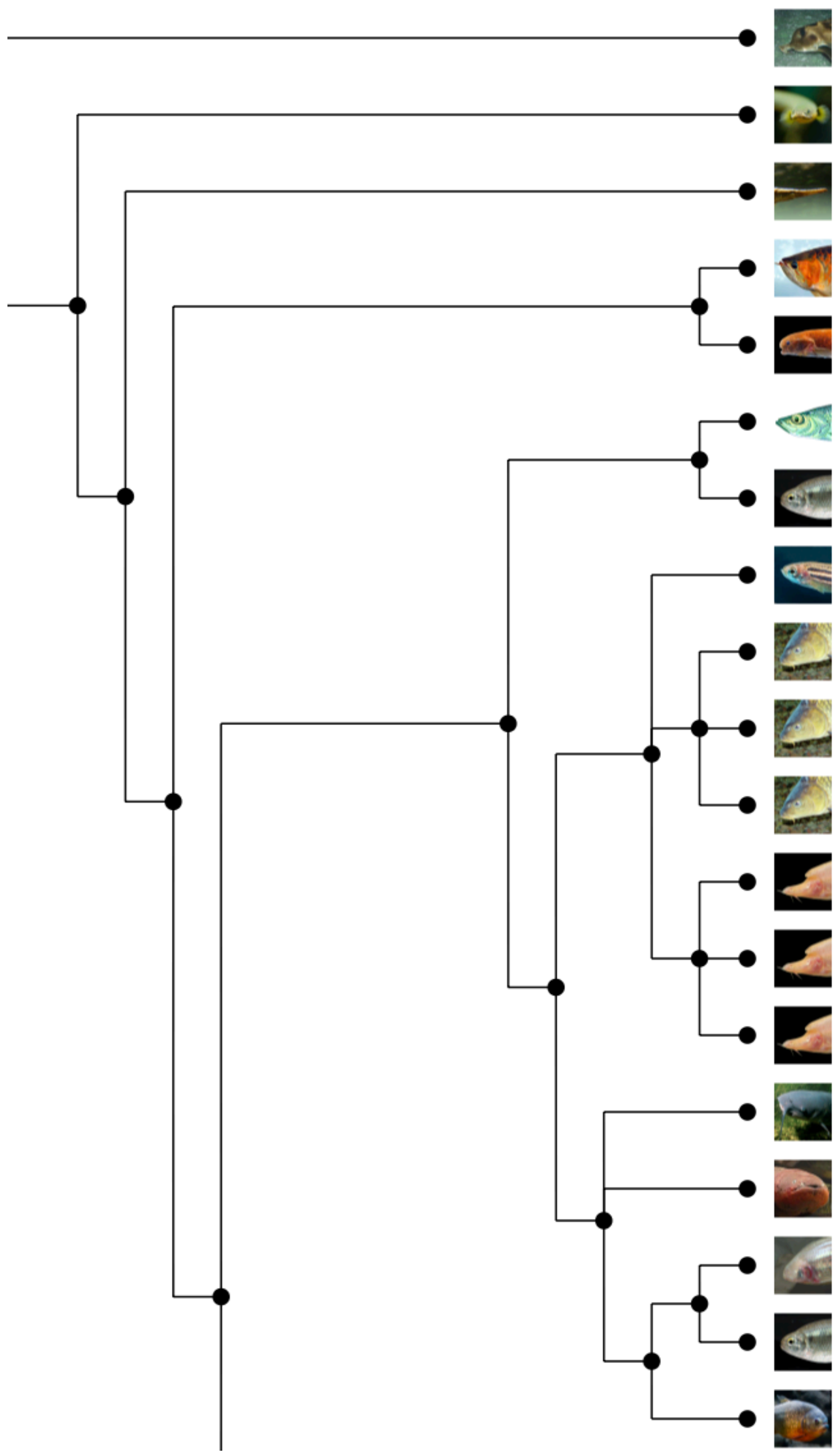

Animal Name	Protocol (2) - mCSM-PPI2 $\Delta\Delta G$	Sequence Identity	# Mutated Residue	Sum Grantham Score
Elephant shark	8.569	58.96	24	1524
Reedfish	6.979	57.92	18	1542
Spotted gar	5.158	58.78	25	1926
Asian bonytongue	7.478	59.23	26	1813
Paramormyrops kingsleyae	NIL	NIL	NIL	NIL
Atlantic herring	7.277	57.91	28	1877
Denticle herring	7.706	59.51	23	1575
Zebrafish	5.835	57.46	22	1333
Common carp hebaored	6.242	56.34	23	1501
Common carp huang he	2.423	59.09	22	1475
Common carp german mirror	5.798	57.57	23	1563
Golden-line barbel	NIL	NIL	NIL	NIL
Horned golden-line barbel	5.435	57.16	23	1493
Blind barbel	3.868	56.45	23	1461
Channel catfish	6.25	57.53	24	1499
Electric eel	5.731	59.07	22	1499
Pachon cavefish	NIL	NIL	NIL	NIL
Mexican tetra	4.348	59.46	22	1656
Red-bellied piranha	NIL	NIL	NIL	NIL

Fishes (2)

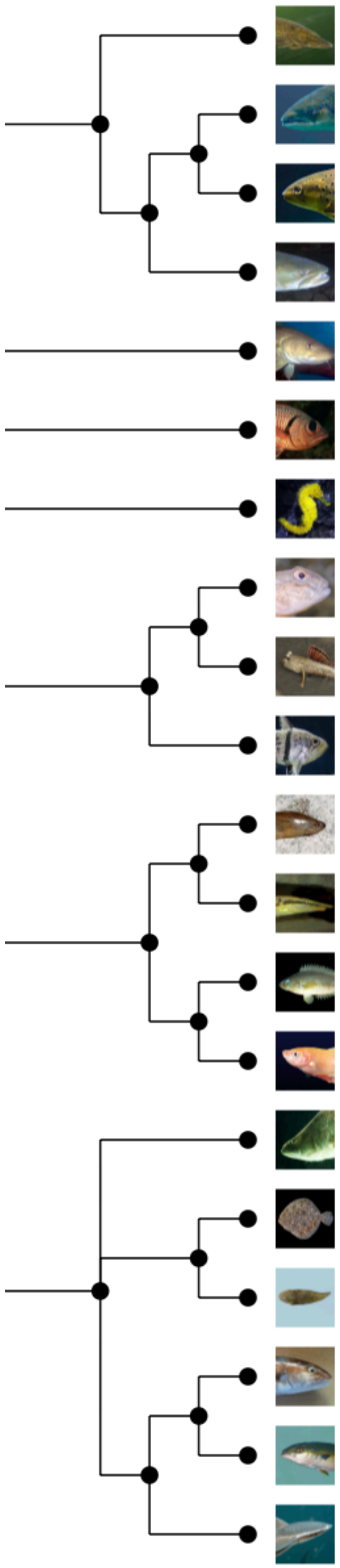

Animal Name	Protocol (2) - mCSM-PPI2 ΔΔG	Sequence Identity	# Mutated Residue	Sum Grantham Score
Northern pike	2.123	58.04	26	1865
Atlantic salmon	NIL	NIL	NIL	NIL
River trout	4.757	57.86	26	1733
Huchen	6.074	56.22	23	1507
Cod	3.852	60.03	24	1597
Pinecone soldierfish	9.666	58.82	21	1566
Tiger tail seahorse	NIL	NIL	NIL	NIL
Round goby	9.83	56.65	27	1644
Periophthalmus magnuspinnatus	NIL	NIL	NIL	NIL
Orbiculate cardinalfish	NIL	NIL	NIL	NIL
Swamp eel	4.295	54.24	22	1596
Zig-zag eel	6.014	57.91	26	1753
Climbing perch	8.16	58.96	26	1648
Siamese fighting fish	3.956	58.89	22	1521
Barramundi perch	3.028	58.86	22	1453
Turbot	3.218	57.36	23	1607
Tongue sole	9.281	57.56	23	1489
Greater amberjack	NIL	NIL	NIL	NIL
Yellowtail amberjack	7.959	58.15	24	1619
Live sharksucker	7.637	57.97	25	1738

Fishes (3)

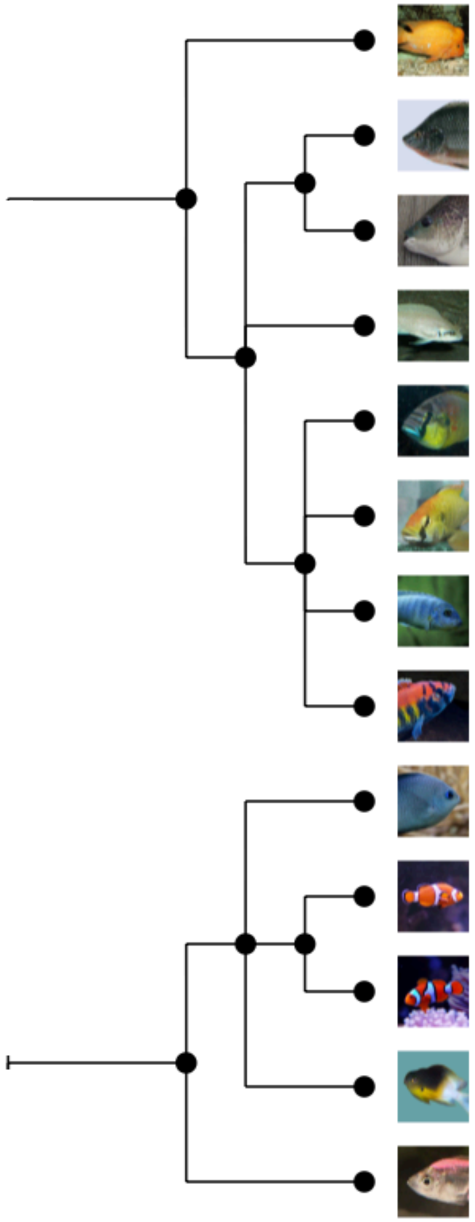

Animal Name	Protocol (2) - mCSM-PPI2 ΔΔG	Sequence Identity	# Mutated Residue	Sum Grantham Score
Midas cichlid	2.188	57.69	23	1538
Nile tilapia	0.664	56.74	20	1364
Blue tilapia	3.864	57.16	21	1387
Lyretail cichlid	3.149	58.07	21	1432
Burton's mouthbrooder	3.556	58.07	21	1387
Eastern happy	3.47	58.07	21	1387
Zebra mbuna	4.075	58.07	21	1387
Makobe Island cichlid	4.95	58.89	21	1387
Spiny chromis	5.419	56.47	26	1736
Clown anemonefish	7.337	58.18	23	1755
Orange clownfish	5.599	56.34	23	1755
Bicolor dameselfish	NIL	64.32	NIL	NIL
Indian glassy fish	6.815	56.27	24	1744

Fishes (4)

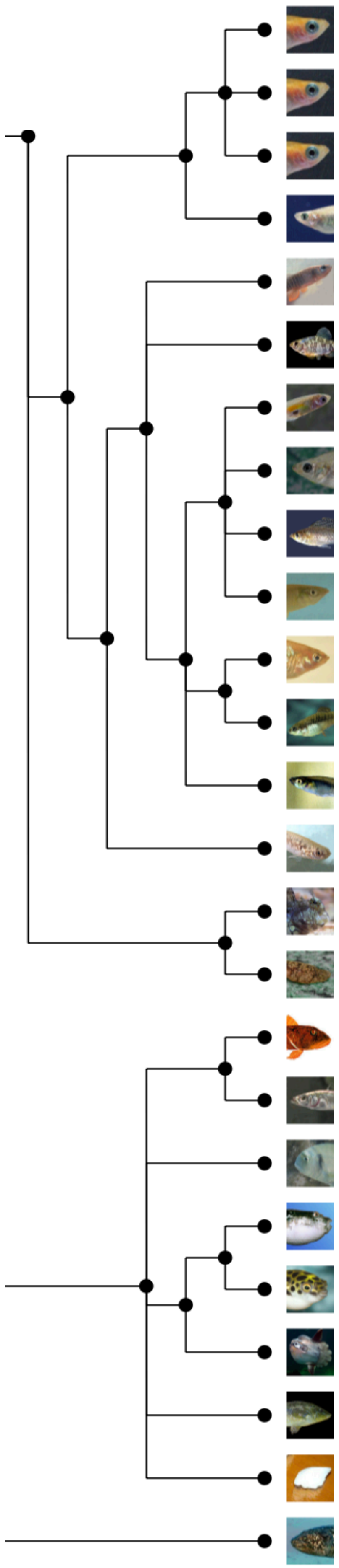

Animal Name	Protocol (2) - mCSM-PPI2 ΔΔG	Sequence Identity	# Mutated Residue	Sum Grantham Score
Japanese medaka HSOK	7.204	57.98	24	1817
Japanese medaka HdrR	NIL	NIL	NIL	NIL
Japanese medaka HNI	5.987	56.94	24	1817
Indian medaka	3.99	55.84	24	1802
Mummichog	7.821	58.36	24	1531
Sheepshead minnow	4.826	58.99	24	1565
Guppy	NIL	NIL	NIL	NIL
Amazon molly	5.66	57.53	25	1617
Sailfin molly	NIL	NIL	NIL	NIL
Shortfin molly	6.512	57.65	25	1617
Platyfish	6.332	58.60	25	1649
Monterrey platyfish	NIL	NIL	NIL	NIL
Western mosquitofish	8.745	58.03	25	1649
Mangrove rivulus	6.228	58.33	23	1763
Jewelled blenny	6.587	56.97	22	1640
Blunt-snouted clingfish	8.498	59.87	26	1835
Channel bull blenny	6.876	56.80	22	1659
Stickleback	4.767	58.11	22	1659
Gilthead seabream	8.471	58.23	23	1480
Fugu	5.871	55.31	23	1684
Tetraodon	5.336	57.20	24	1814
Ocean sunfish	5.064	58.85	23	1633
Ballan wrasse	8.614	56.18	25	1891
Large yellow croaker	3.538	58.99	23	1435
Coelacanth	7.845	60.96	25	1744
